# The Combination Effects of LiCl and the Active Leflunomide Metabolite, A771726, on Viral-Induced Interleukin 6 Production and EV-A71 Replication

**DOI:** 10.1371/journal.pone.0111331

**Published:** 2014-11-20

**Authors:** Hui-Chen Hung, Shin-Ru Shih, Teng-Yuan Chang, Ming-Yu Fang, John T.-A. Hsu

**Affiliations:** 1 Institute of Biotechnology and Pharmaceutical Research, National Health Research Institutes, Miaoli, Taiwan; 2 Department of Medical Biotechnology & Laboratory Science, Chang Gung University, Tao-Yuan, Taiwan; 3 Clinical Virology Laboratory, Department of Clinical Pathology, Chang Gung Memorial Hospital, Tao-Yuan, Taiwan; 4 Research Center for Emerging Viral Infections, Chang Gung University, Taoyuan, Taiwan; 5 Department of Biological Science and Technology, National Chiao Tung University, Hsinchu, Taiwan; The Scripps Research Institute, United States of America

## Abstract

Enterovirus 71 (EV-A71) is a neurotropic virus that can cause severe complications involving the central nervous system. No effective antiviral therapeutics are available for treating EV-A71 infection and drug discovery efforts are rarely focused to target this disease. Thus, the main goal of this study was to discover existing drugs with novel indications that may effectively inhibit EV-A71 replication and the inflammatory cytokines elevation. In this study, we showed that LiCl, a GSK3β inhibitor, effectively suppressed EV-A71 replication, apoptosis and inflammatory cytokines production (Interleukin 6, Interleukin-1β) in infected cells. Furthermore, LiCl and an immunomodular agent were shown to strongly synergize with each other in suppressing EV-A71 replication. The results highlighted potential new treatment regimens in suppressing sequelae caused by EV-A71 replication.

## Introduction

Human *enteroviruses* A belong to the family *Picornaviridae* [1], which has more than 23 serotypes, including coxsackievirus A2 (CV-A2), CV-A3, CV-A4, CV-A5, CV-A6, CV-A7, CV-A8, CV-A10, CV-A12, CV-A14, CV-A16, enterovirus A71 (EV-A71), EV-A76, EV-A89, EV-A90, EV-A91, EV-A114, EV-A119 and the simian enteroviruses EV-A92, SV19, SV43, SV46 and baboon enterovirus A13 (BA13) [2]. EV-A71 is one of the most important neurotropic enteroviruses associated with severe central nervous system (CNS) inflammation [3], [4]. In addition to severe meningitis during acute infection, children recovering from EV-A71 infection were recently shown to have a higher incidence of mental disorders, such as hyperactivity- and impulsivity-related syndromes [5], [6].

High levels of proinflammatory cytokines, such as Interleukin 6 (IL-6), Tumor necrosis factor α (TNF-α) and Interleukin-1β (IL-1β), have been found in EV-A71 patients with pulmonary edema (PE) and encephalitis [6], [7]. A recent study has revealed an association between the levels of proinflammatory cytokines in the cerebrospinal fluid (CSF) and the severity of EV-A71 brain stem encephalitis (BE) [8]. In particular, the CSF levels of IL-6 were shown to be closely correlated with clinical severity in EV-A71 patients and a neonatal mouse model of infection [9], [10]. Intravenous immune globulin (IVIG) and milrinone were found to modulate cytokine network in severe EV-A71-infected patients. Based on these results, cytokine storm was hypothesized to play a prominent role in the progression of severe EV-A71 disease, including the associated tissue damage and immunopathology [10], [11]. Thus, it is important to identify effective therapeutics to modulate the inflammatory responses during EV-A71 infection.

Little is known regarding the molecular mechanisms underlying the cytokine responses to EV-A71 infection in neural cells. We have previously identified 157 genes whose mRNA levels were substantially different in EV-A71-infected human neural SF268 cells utilizing cDNA microarray analysis [12]. A subset of cytokines was found to be induced in EV-A71-infected neural cells, including RANTES, IL-8, and chemokine receptor 5. Recently, genome-wide RNAi screening datasets suggested that several host factors involved in the altered cytokine network in EV-A71-infected cells may be effective drug targets for the development of antiviral therapeutics. Upon EV-A71 infection, initial PI3K/Akt signaling followed by MAPK/ERK activation and subsequent GSK3β inactivation is hijacked by EV-A71 as a potential mechanism to delay host cell apoptosis [13]. Furthermore, GSK3β is a potent regulator of the homeostasis between the pro- and anti-inflammatory cytokine levels in the CNS [14]. Thus, the possible involvement of the PI3K-Akt-GSK3β signaling axis in EV-A71 replication in neural cells indicates that this pathway may be a druggable target.

In this study, we hypothesized that signal transduction networks could be pharmacologically perturbed to modulate EV-A71 replication and the subsequent cytokine responses in the neural cell system. The results of this study suggest that existing anti-viral target could be used as novel treatments for EV-A71.

## Materials and Methods

### Cells, viruses, and reagents

African green monkey kidney cells (Vero cells: ATCC-CCL-81) were purchased from the American Type Culture Collection (ATCC) and cultured in Minimum Essential Medium (Invitrogen, Grand Island, NY) supplemented with 10% FBS. A human CNS glioblastoma cell line (SF-268) was obtained from the Culture Collection Center, Hsin-Chu, Taiwan and cultured in RPMI 1640 medium (Invitrogen, Grand Island, NY). The SY-SH5Y cell line was kindly provided by Dr. Jyh Lyh Juang at the National Health Research Institutes (NHRI) of Taiwan and cultured in Minimum Essential Medium (Invitrogen, Grand Island, NY) supplemented with 10% FBS [15]. EV-A71-2231-TW was isolated during the 1998 outbreak and was supplied by the Clinical Virology Laboratory of Chang Gung Memorial Hospital, Taiwan [16]. Virus titers were measured with plaque assays using Vero cells. SB203580, AR-A04418 and Wortmannin were purchased from Calbiochem (EMD Biosciences, Inc., La Jolla, CA).MTS(tetrazolium compound [3-(4,5-dimethylthiazol-2-yl)-5-(3-carboxymethoxyphenyl)-2-(4-sulfophenyl)-2*H*-tetrazolium, inner salt]), PMS (phenazine methosulfate), Dimethyl Sulfoxide (DMSO), LY294002 and LiCl were purchased from Sigma-Aldrich Corporation (Saint Louis, MO). A771726 was purchased from Santa Cruz Biotechnology, Inc. (San Diego, CA, USA).

### Virus yield assay

A total of 5×10^5^ SF268 cells were seeded into 6-well plates and allowed to reach confluence. Then, the cells challenged with virus (moi 0.5) and exposed to various concentrations of compounds after viral adsorption for 1 hr. After 48 h, the culture media and cell lysates were collected following freeze-thaw cycles and subjected to virus titration using plaque-forming assays, as previously described [17].

### Western blotting

The cell lysates collected and lysed with lysis buffer (150 mM NaCl, 1% CA630, and 50 mM Tris-base, pH 8.0) to detect the target proteins. Proteins in sample buffer were subjected to SDS-PAGE and electroblotted onto Hybond ECL membranes (GE Healthcare, Uppsala, Sweden). Blocking and incubation with antibodies were performed using 0.05% Tween 20 and Tris-buffered saline. The western blot membranes were incubated with a 1∶2000 dilution of an anti-GSK3β rabbit polyclonal Ab or an anti-Bcl-2 rabbit polyclonal Ab at either 4°C overnight or room temperature for 2 h. Primary antibodies were purchased from Cell Signaling Technology Inc. (Danvers, MA, USA). The blots washed with TBS-Tween for 1 h and then incubated with an HRP-conjugated anti-rabbit IgG. Secondary antibody was purchased from Santa Cruz Biotechnology, Inc. (San Diego, CA, USA). An anti-EV-A71-specific monoclonal antibody MAB979 (IgG1 subclass) was obtained from Chemicon-Millipore, Inc. (Temecula, CA, USA).

### Cytotoxicity assay

Cell viability was determined by MTS assay [18], as described by Hung *et al.* [19]. SF268 cells were grown (7000 cells/well) in 96-well plate for 24 h. The medium was replaced with fresh medium with 2% fetal bovine serum (FBS) and a fourfold serial dilution of the test compound (each dilution in triplicate). After 72 h, The culture medium were removed and added 100 µl phenol red-free medium containing MTS and PMS mixture solution, the plate was incubated at 35°C for 30 min to 1 h. The optical density was measured at OD490 nm in ELISA reader. The 50% cytotoxic concentration (CC_50_) was determined as the concentration of the compound at which cell viability was reduced to 50%. Data were analyzed using GraphPad Prism 5.0 software.

### Detection of pro-inflammatory IL-6 mRNA and protein in EV-A71-infected cells

A total of 5×10^5^ SF268 cells were seeded into 6-well plates and allowed to reach confluence. Then, the cells were challenged with virus (moi 0.5). Various concentrations of compounds were added after viral adsorption for 1 hr. At 48 h post-infection, total RNA was extracted from cells using the TRIzol reagent (Invitrogen, Carlsbad, CA, USA) and subjected to reverse transcription PCR (RT-PCR) as previously described [17]. The sequences of the primers used for Q-PCR were as follows:

IL-6 forward primer: 5′-AGGAGACTTGCCTGGTGAAA-3′, and

IL-6 reverse primer: 5′-CAGGGGTGGTTATTGCATCT-3′.

IL-1β forward primer: 5′-ACAGATGAAGTGCTCCTTCCA-3′.

IL-1β reverse primer: 5′-GTCGGAGATTCGTAGCTGGAT-3′.

The results were normalized to the RNA level of the reference gene glyceraldehyde-3-phosphate dehydrogenase (GAPDH)

GAPDH forward primer, 5′-GAAGGTGAAGGTCGGAGT-3′,

GAPDH reverse primer, 5′-GAAGATGGTGATGGGATTTC-3′.

The IL-6 protein level was measured using an enzyme-linked immunosorbent kit from eBioscience (San Diego, CA, USA) according to the manufacturer's instructions.

### Detection of virus-induced cytopathic effects (CPEs)

The anti-EV-A71 activity of A771726 was measured by evaluating the inhibition of the virus-induced cytopathic effect. Twenty-four-well tissue culture plates were seeded with 500 µL of 2×10^5^ SF268 cells/mL in DMEM containing 10% FBS. The cells were incubated for 18 to 24 h at 37°C, and challenged with virus (moi 0.5), and exposed to various concentrations of A771726 and uridine in DMEM containing 2% FBS. After 1 h of viral adsorption, the infected cells were overlaid with 50 µL of DMEM with 2% FBS and 0.5% DMSO and incubated at 37°C for 72 h. At the end of the incubation, the cells were fixed with formaldehyde and stained with 0.1% crystal violet as described in a previous report [17].

## Results

### Inhibition of EV-A71 replication and cell death through the pharmacological inhibition of glycogen synthase kinase-3 (GSK3) activity

**Figure 1:**
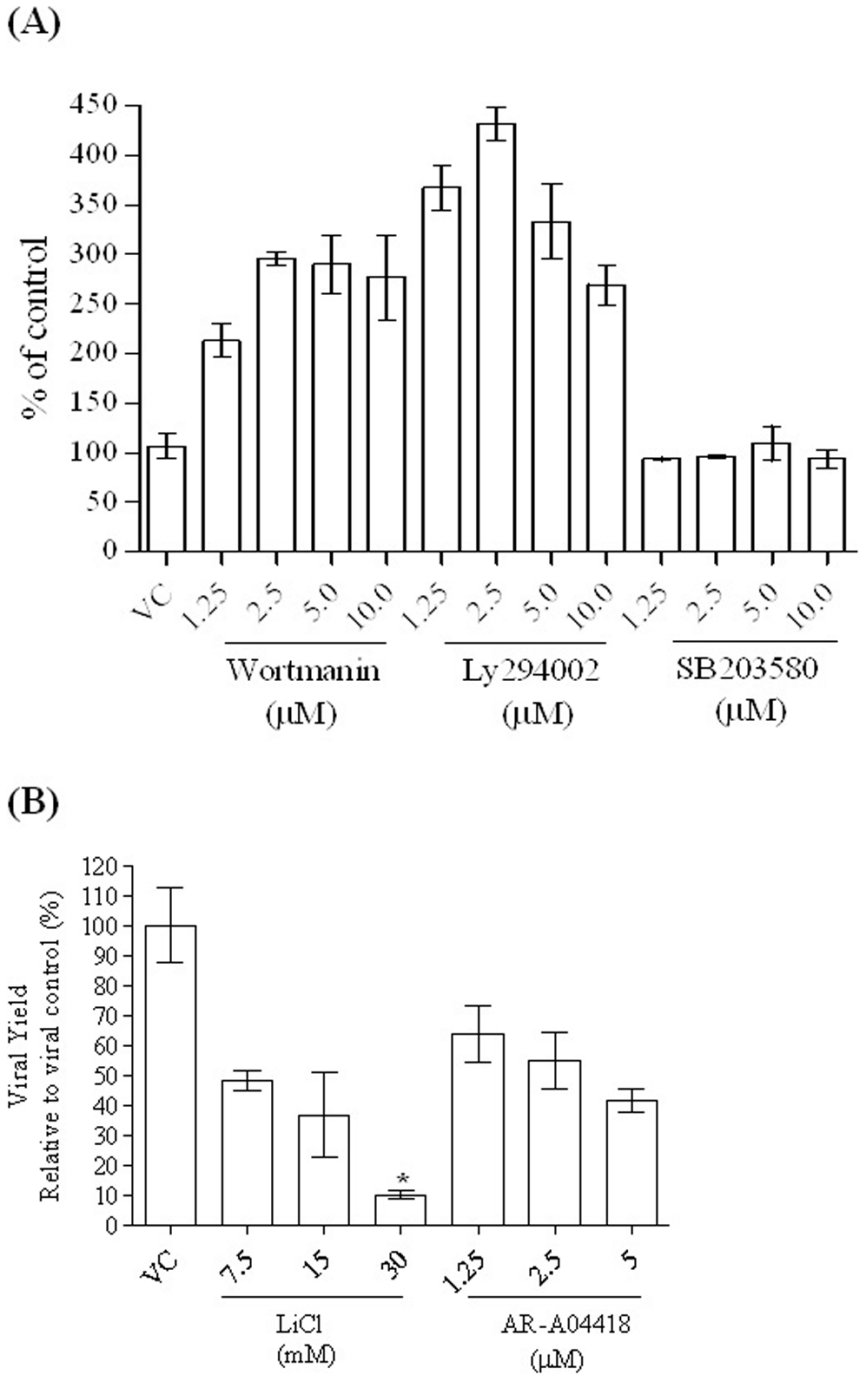
Pharmacological interventions targeting PI3K signaling affect EV-A71 replication in SF268 neural cells. The effect of PI3K (LY294002 and wortmannin), P38 MAPK (SB203580) (A) and GSK3β (AR-A014418 and LiCl) (B) inhibitors as pharmacological interventions on the virus yields of EV-A71-infected cells was assessed. SF268 cells were infected with EV-A71 (moi 0.5), and various concentrations of compounds were added to the infected cells after 1 h viral adsorption. At 48 h post-infection, the culture supernatants and cell lysates were collected for virus titration using plaque assays. Data are displayed as mean ± s.e.m. from at least two independent experiments performed in duplicates.

PI3K signaling mediates a variety of intracellular responses. Several studies have demonstrated that viruses can modulate the cytokine and apoptotic process of host cells in regulating PI3K-Akt pathway [20], [21]. To further characterize the signal pathway involved in EV-A71-induced IL-6 expression and virus replication in SF268 cells, we firstly investigated whether EV-A71 replication in SF268 cells could be pharmacologically perturbed by inhibitors against PI3K (LY294002 and wortmannin), GSK3β (AR-A014418 and LiCl) and PI3K/p38 MAPK kinase (SB203580). SF268 cells were infected with EV-A71, and various concentrations of compounds were added to the infected cells after virus adsorption stage. At 48 h post-infection, the culture supernatants and cell lysates were collected for virus titration using plaque assays. Treatment of confluent SF268 neural cells with either LY294002 or wortmannin enhanced the replication of EV-A71 (Fig 1(A)). Whereas, p38 MAPK inhibitor SB203580 did not inhibit viral yield (Fig 1(A)). Subsequently, we investigated whether EV-A71 replication in SF268 cells could be affected by AR-A014418 and LiCl. Infected SF268 neural cells treated with AR-A014418 or LiCl yielded lower viral loads (Fig. 1(B)). Treatment with 15 mM LiCl resulted in a 50% reduction in the virus yield. At 30 mM, LiCl caused an approximately 85% reduction in the virus yield of EV-A71-infected SF268 cells. The cytotoxicity of inhibitors and LiCl in this study were monitored by MTS assay (Fig. S1(A) and Fig. S1(B)); results indicate that the 50% cytotoxic concentration (CC_50_) value was>60 mM (Fig. S1(B)). Thus, the antiviral effects of lithium may be physiologically achieved.

**Figure 2:**
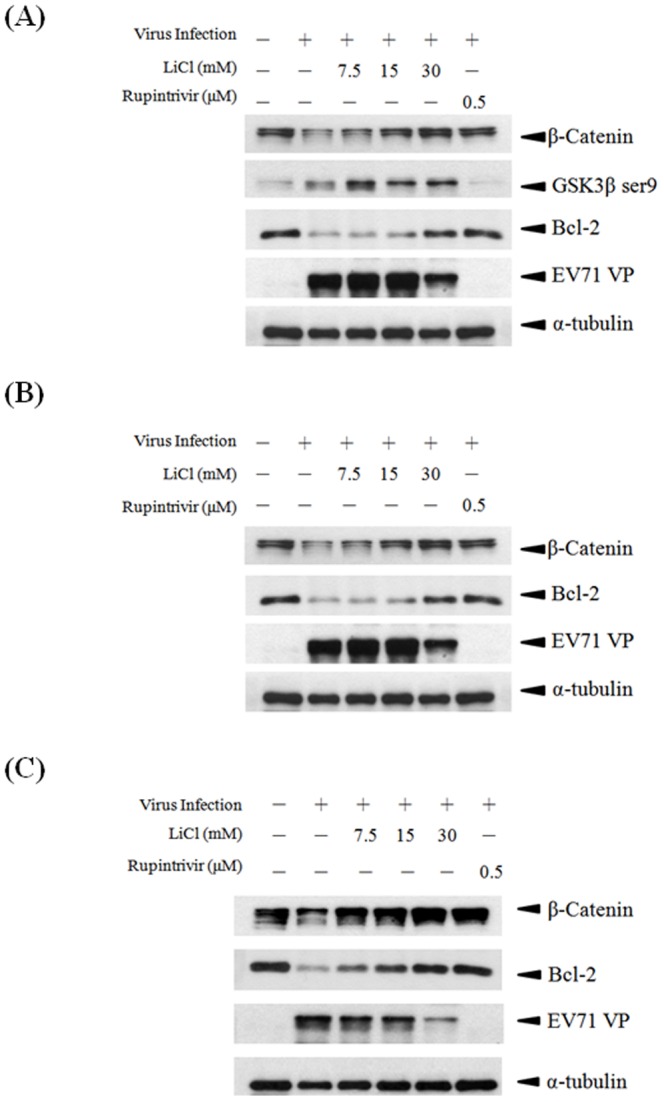
LiCl inhibits EV-A71 replication in SF268 cells (A), (B) and SH-SY5Y cells (C). Cells were infected with EV-A71 (moi 0.5), and various concentrations of LiCl were added to the infected cells after viral adsorption time. Lysates collected At 8 h (**A**) and 72 h (**B, C**) post-infection were analyzed with immunoblotting. Rupintrivir was employed as a positive control because it can effectively inhibit EV-A71 replication. Measurements were made in three independent experiments.

The level of Ser9 phosphorylation in GSK3β was enhanced by LiCl in a dose-dependent manner (Fig. 2(A)), indicating that GSK3β was effectively targeted. In addition to reducing the yield of viral progeny in EV-A71-infected cells, LiCl also inhibited viral protein (VP1) synthesis in SF268 cells (Fig. 2(A)). A viral 3C protease inhibitor, rupintrivir, was employed as a positive control that can effectively inhibit EV-A71 replication [22]–[24]. Because GSK3β mediates its apoptotic effects through Bcl-2 and β-catenin, the effects of LiCl on Bcl-2 and β-catenin in EV-A71-infected cells were then examined. The Bcl-2 level was down-regulated in EV-A71-infected SF268 cells compared with that in uninfected cells (lanes 1 and 2, Fig. 2(B)). The Bcl-2 and β-catenin levels in EV-A71-infected cells were restored to different degrees upon LiCl treatment (lanes 3, 4, and 5). A similar phenomenon was also observed in EV-A71-infected SH-SY5Y neuroblastoma cells treated with LiCl after virus adsorption stage (Fig. 2(C)).

### LiCl decreased pro-inflammatory cytokine IL-6 and IL-1β production in EV-A71-infected cells

**Figure 3:**
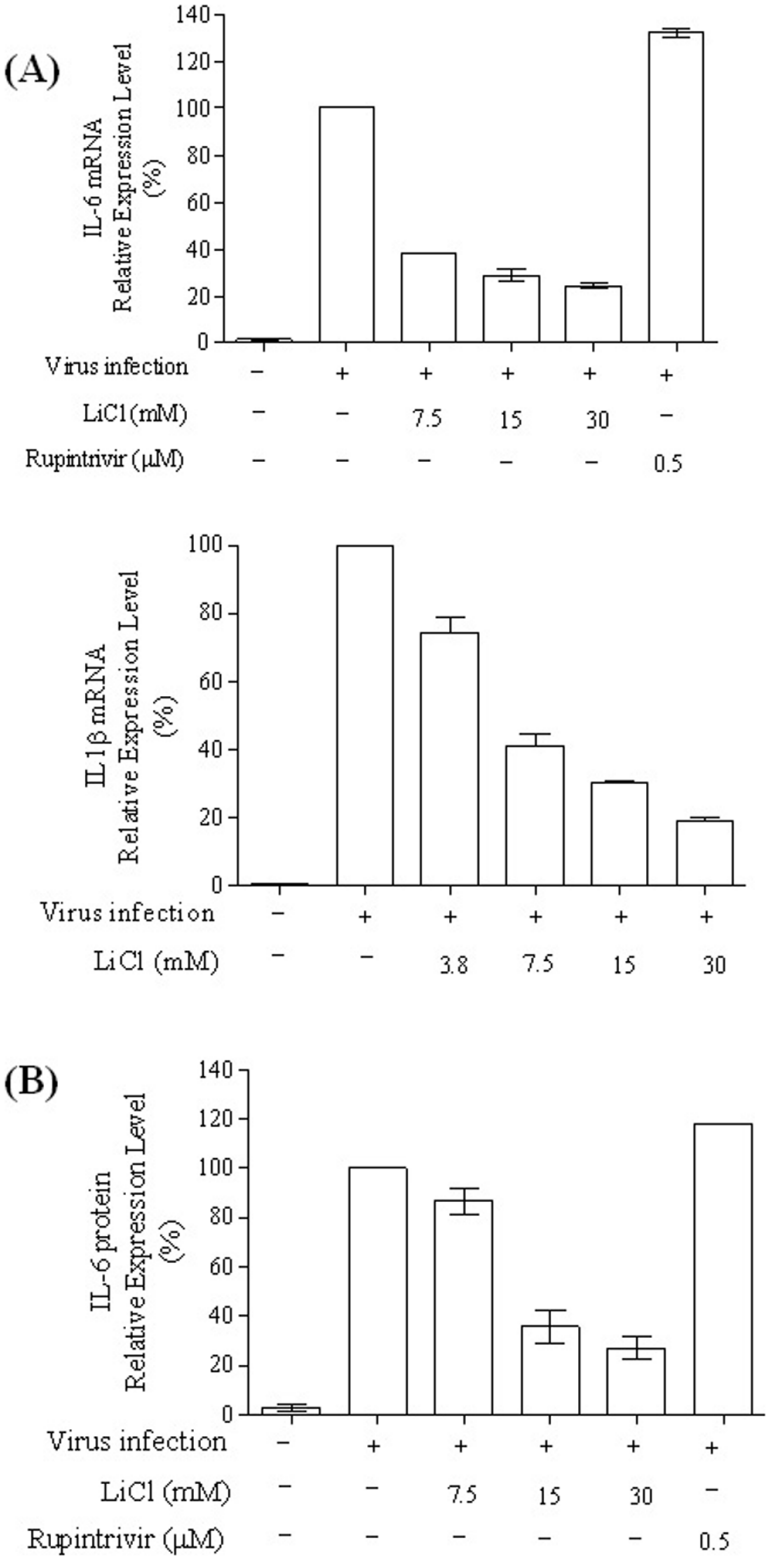
LiCl suppresses pro-inflammatory IL-6 and IL-1β mRNA expression (A) and IL-6 protein expression (B) in EV-A71-infected SF268 cells. SF268 cells were infected with EV-A71 (moi 0.5), and various concentrations of LiCl were added to the infected cells after viral adsorption time. At 48 h post-infection, the cell lysates were collected for quantification of the IL-6 and IL-1β mRNA. In a parallel experiment, the IL-6 protein levels in cell culture supernatant were measured using enzyme-linked immunosorbent assay. Data are the mean ± s.e.m. from at least three parallel measurements per experiment.

Because GSK3β plays an important role in controlling the pro-inflammatory cytokine response in host cells [25], we sought to determine whether inhibiting this pathway with LiCl could lead to a reduction in the IL-6 and IL-1β expression level in EV-A71-infected cells. EV-A71-infected cells treated with LiCl at various concentrations were examined for IL-6 and IL-1β production at 48 h post-infection. The results demonstrated that LiCl reduced the IL-6 and IL-1β mRNA (Fig. 3(A)) and IL-6 protein levels (Fig. 3(B)) in EV-A71-infected cells in a dose-dependent manner (Fig. 3). It is worth noting that rupintrivir, a 3C protease inhibitor, did not inhibit IL-6 expression in EV-A71-infected cells, even though it was able to effectively inhibit virus replication. In contrast, we also tested p38 kinase inhibitor (SB203580) on IL-6 expression in EV-A71-infected cells and results showed that the p38 MAPK inhibitor, SB203580, did not inhibit viral yield but did exert inhibitory effect on IL-6 expression in infected in infected cells (Data not shown).

### Synergistic anti-EV-A71 activity of the combination of LiCl and immunosuppressive agent A771726 in neural cells

**Figure 4:**
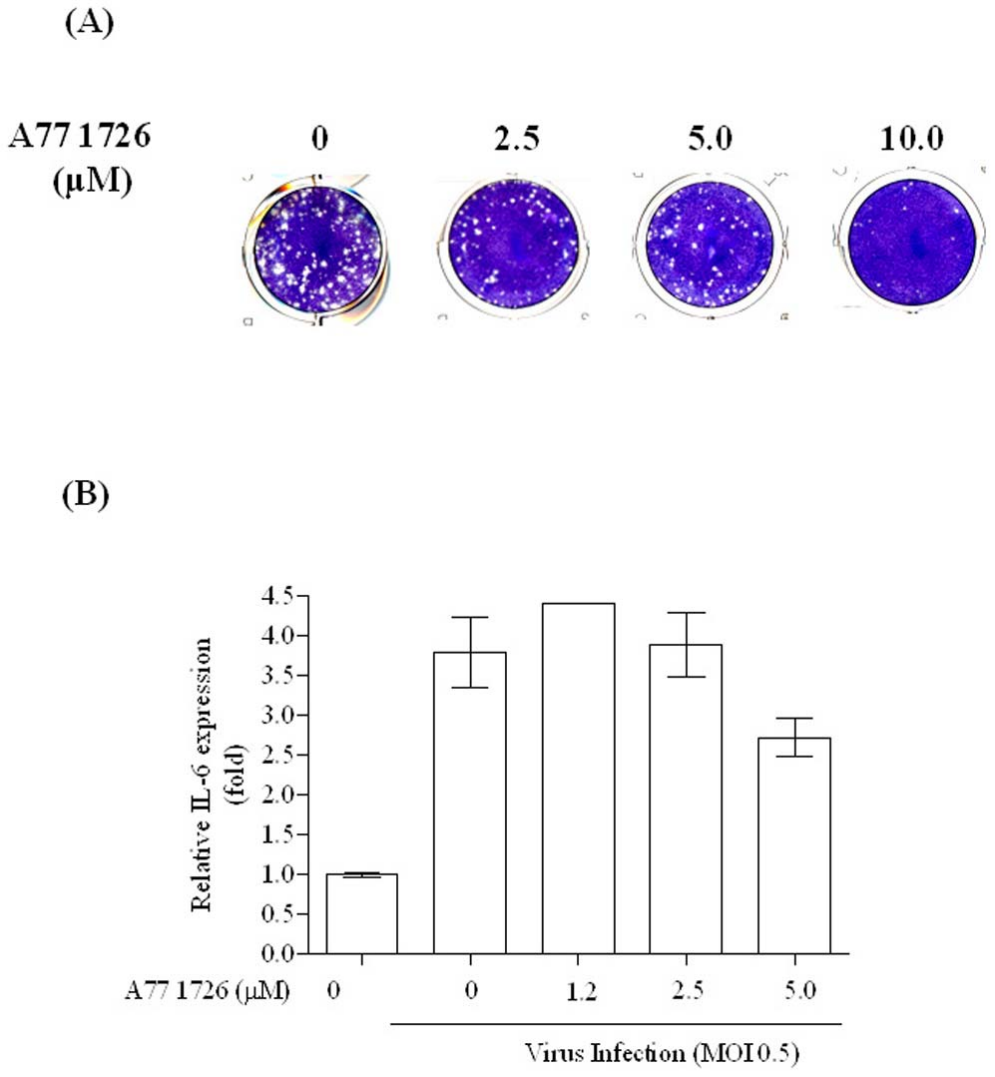
The active metabolite of Leflunomide, A771726, suppress virus production (A) and the expression of the pro-inflammatory cytokine IL-6 (B) in EV-A71-infected SF268 cells. SF268 cells were infected with EV-A71 (moi 0.5), and various concentrations of A771726 were added to the infected cells after viral adsorption time. At 48 h post-infection, the culture supernatants and cell lysates were collected for virus titration (A). In a parallel experiment, the IL-6 protein levels in cell culture supernatant were measured using ELISA (B). Each virus stock to be titered is serially diluted in10-fold series (dilution fold 10^−4^) and added to Vero cells. Data are the mean ± s.e.m. from at least two parallel measurements per experiment.

We then sought to combine drugs of distinct mechanisms and attempt to maximize their anti-EV71 effects. Leflunomide (Arava) and its active metabolite A771726 have shown interesting antiviral and immunosuppressive effects [26]. A771726 is neuroprotective in a central nervous system disease [27]. The cytotoxicity of A771726 was monitored by MTS assay; results indicate that the CC_50_ value was 34.3 µM (Fig. S1(B)).We therefore determined whether A771726 interfered with the host signaling pathway in EV-A71-infected cells. Cells were infected with EV-A71 and treated with various concentrations of A771726. At 48 h post-infection, culture supernatants and cell lysates were collected for measurements of the virus titer and IL-6 levels. As shown in Fig. 4(A), A771726 effectively inhibited EV-A71 replication in neural cells. The levels of IL-6 in infected cells were 3- to 4-fold higher than those in uninfected cells. A771726 at 5 µM inhibited more IL-6 production in EV-A71-infected cells (Fig. 4(B)).

We next evaluated whether the combination of A771726 and LiCl exerted synergistic, additive or antagonistic anti-EV-A71 effects. Inhibition of EV71 replication was evaluated in CPE test involving treatment with serial dilutions of A771726 (0, 0.625, 1.25, 2.5, 5 and 10 µM) or LiCl (0, 1.25, 2.5, 5, 10, 20 and 40 mM), or with a combination of the 2 compounds at fixed 2∶1 or 4∶1 ratios (Table 1). CalcuSyn analysis was employed to provide combination index (CI) values to determine potential drug additivity in Table 1 [28]. Results revealed statistically synergistic anti-EV71 effects at 50% inhibition levels.

**Table 1:** Effects of A771726 in combination with LiCl on EV71- induced cytopathic effect.

ED	CI with the following A771726/LiCl ratios	
	2∶1	4∶1
50%	0.49	0.24
75%	0.31	0.29
90%	0.20	0.37

CalcuSyn analysis provides combination index (CI) values to determine potential drug additivity in this Table. Inhibition of EV71 replication was evaluated in CPE test involving treatment with serial dilutions of A771726 (0, 0.625, 1.25, 2.5, 5 and 10 µM) or LiCl (0, 1.25, 2.5, 5, 10, 20 and 40 mM), or with a combination of the 2 compounds at fixed 2∶1 or 4∶1 ratios. The results show that A771726 combined with LiCl is synergistic, with CI values ranging from 0.20 to 0.49.

The virus yield in the culture supernatant was estimated, results showed that the virus yield was dramatically decreased in infected cells treated with 15 mM LiCl in combination with A771726 (Fig. S2(A)). We also evaluated the effects of LiCl and A771726 combination on EV-A71-induced IL-6 production in SF268 cells. A 40–50% reduction in the IL-6 level was observed when infected cells were treated with A771726 at 5 µM or LiCl at 15 mM when used alone (Fig. S2(B)). Strikingly, an approximately 90–95% reduction in the IL-6 level was observed when infected cells were treated with a combination of A771726 at 1.2 µM and LiCl at 15 mM. The level of EV-A71-induced CPE and viral protein synthesis were also significantly attenuated in infected cells by treatment with A771726 combined with low dose of LiCl (Fig. S2(C)).

## Discussion

Understanding the interplay between viruses and host cells is essential for the discovery of effective antiviral therapies. Because EV-A71 infections may cause severe neurological sequelae, we used CNS-derived cells in this study to investigate whether EV-A71 replication in neural cells could be attenuated with existing medicines. Coyne *et al.* reported that Akt family restrict Enterovirus replication in HBMECs [29]. The study used pharmacological inactivation of Akt by Akt1/Akt2 inhibitor and this led to increased enteroviral infection. Furthermore, related experiments found that rapamycin and Akt2 siRNA dramatically increased coxsackie virus B3 (CVB3) replication in primary HBMECs. In this study, we identified the requirement for Akt in antiviral defense occurs via Akt-GSK3β signal pathway for EV-A71 replication. The GSK3β inhibitors AR-A014418 and LiCl were shown to reduce the virus yield of EV-A71.

LiCl was further examined because LiCl is a frequently prescribed drug in the modern pharmacopoeia [30]–[32]. Related studies reported that GSK3β inhibition with LiCl at concentrations up to 30 mM during CVB3 infection only led to a decrease in progeny virus but had no effect on viral protein synthesis [33]. In contrast, the treatment of EV-A71-infected cells with LiCl resulted in reductions in both viral protein synthesis and the yield of released viral progeny. We also found that EV-A71-induced apoptosis was inhibited by LiCl in neural cells. The level of the anti-apoptotic protein Bcl-2 was greatly reduced in EV-A71-infected cells, and treatment with LiCl restored Bcl-2 to the normal level.

The importance of controlling the cytokine network in severely ill EV-A71-infected patients using immunoglobulin (IVIG) or milrinone has been highlighted in a recent review [11]. Leflunomide has been approved for the treatment of active rheumatoid arthritis (RA). Leflunomide has a number of effects, including cytokine-driven immunosuppressive activity [34], the inhibition of dihydroorotate dehydrogenase (DHODH) [35], the inhibition of tyrosine kinases [36], the reduction of inflammatory responses [37], and antiviral activity against a number of different viruses [38]–[43]. Pyrimidine metabolism involving DHODH is required for the replication of some viruses [44]. The results of this study demonstrate that the active leflunomide metabolite, A771726, likely impairs the de novo pyrimidine synthesis required for the EV71 replication cycle because the anti-EV71 effects were reversed by the addition of uridine (Fig. S3). In the present study, we also sought to determine whether immune-modulating agents had synergistic effects when used in combination with LiCl, and we found that leflunomide and its active metabolite A771726 suppressed virus production and pro-inflammatory IL-6 and IL-1β expression in EV-A71-infected SF268 cells. Several lines of evidence indicate that IL-6 is an important factor that is closely correlated with clinical severity [6], [9], [45]. IL-6 was found as an indicator of EV-A71 encephalitis with pulmonary edema [45]. High levels of IL-6 in a neonatal mouse model upon EV-A71 infection were shown to result in severe tissue damage and eventual death [10].

It is clear that host signaling pathways play important roles in virus replication. In this study, we found that both EV-A71 replication and EV71-A71-induced IL-6 production in neural cells were effectively subdued by LiCl. Further investigations are needed to explore if lithium and A771726 also affects other viral or cellular functions that are important for EV-A71 replication. The results from this study have highlighted the possibility of targeting the host inflammatory response and cell factors involved in virus replication to develop new treatment regimens in suppressing severe EV-A71 replication.

## Supporting Information

Figure S1 **The cytotoxicity effect of PI3K (LY294002 and wortmannin), P38 MAPK (SB203580) (A), GSK3β (LiCl (B) and AR-A014418(C)) and A771726 (C) in this study.** Cytotoxicity assays were performed as described in the materials and methods section. Data are displayed as mean ± s.e.m. of triplicate measurements and are representative of three independent experiments.
(TIF)

Figure S2 **The effects of the combination of LiCl and A771726 on virus yield (A), IL-6 expression levels (B) and viral protein synthesis (C) in EV-A71-infected SF268 cells.** SF268 cells were infected with EV-A71 (moi 0.5), and various concentrations of LiCl and A771726 were added to the infected cells for 48 h after infection. Assays were performed as described in the materials and methods section. Data are the mean ± s.e.m. from at least three parallel measurements per experiment. Symbol indicates significant differences as determined by one way ANOVA: *P<0.05, **P<0.001 *: compared to the percent of infected cells by treatment with A77172.
(TIF)

Figure S3 **Inhibitory effects of A771726 on EV71-induced CPEs and the reversal of EV71-induced CPEs by uridine.** Cells were infected with EV-A71 (moi 0.5), and various concentrations of A771726 and uridine were added to the infected cells. SF268 cells were all lysed at 72 h after EV-A71 infection, as shown in the VC (virus control) column. Measurements were made in three independent experiments.
(TIF)
