# Supplementary figures and images for: The Combination Effects of LiCl and the Active Leflunomide Metabolite, A771726, on Viral-Induced Interleukin 6 Production and EV-A71 Replication
Source: PLoS One. 2014 Nov 20;9(11):e111331. doi: 10.1371/journal.pone.0111331 (PMC4239034; doi:10.1371/journal.pone.0111331)

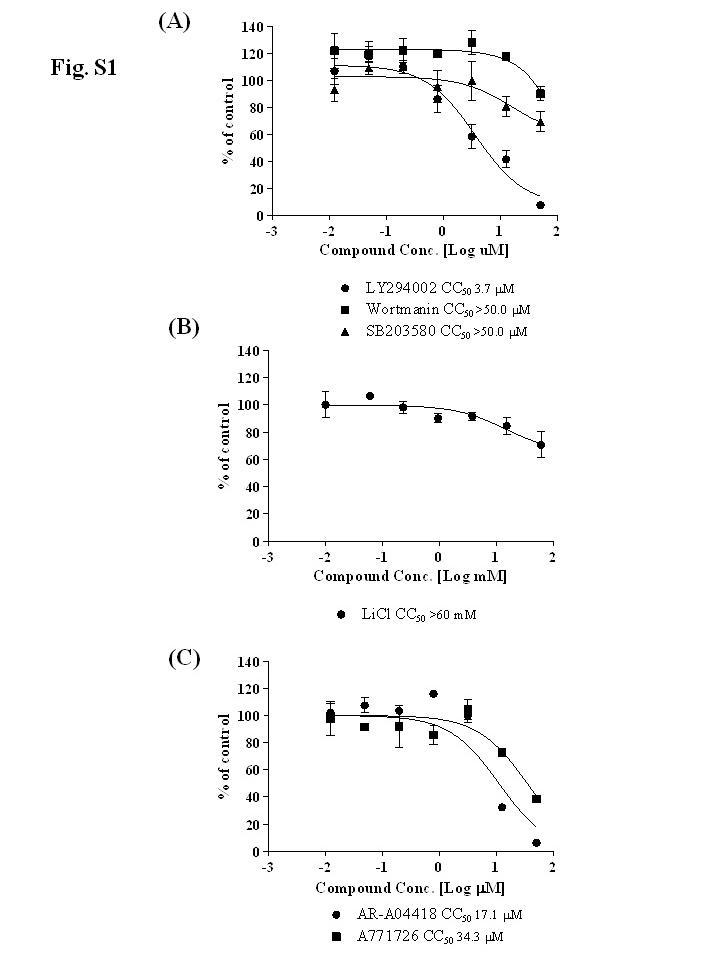

Supplement: Figure S1 — The cytotoxicity effect of PI3K (LY294002 and wortmannin), P38 MAPK (SB203580) (A), GSK3β (LiCl (B) and AR-A014418(C)) and A771726 (C) in this study. Cytotoxicity assays were performed as described in the materials and methods section. Data are displayed as mean ± s.e.m. of triplicate measurements and are representative of three independent experiments. (TIF) [file pone.0111331.s001.tif]

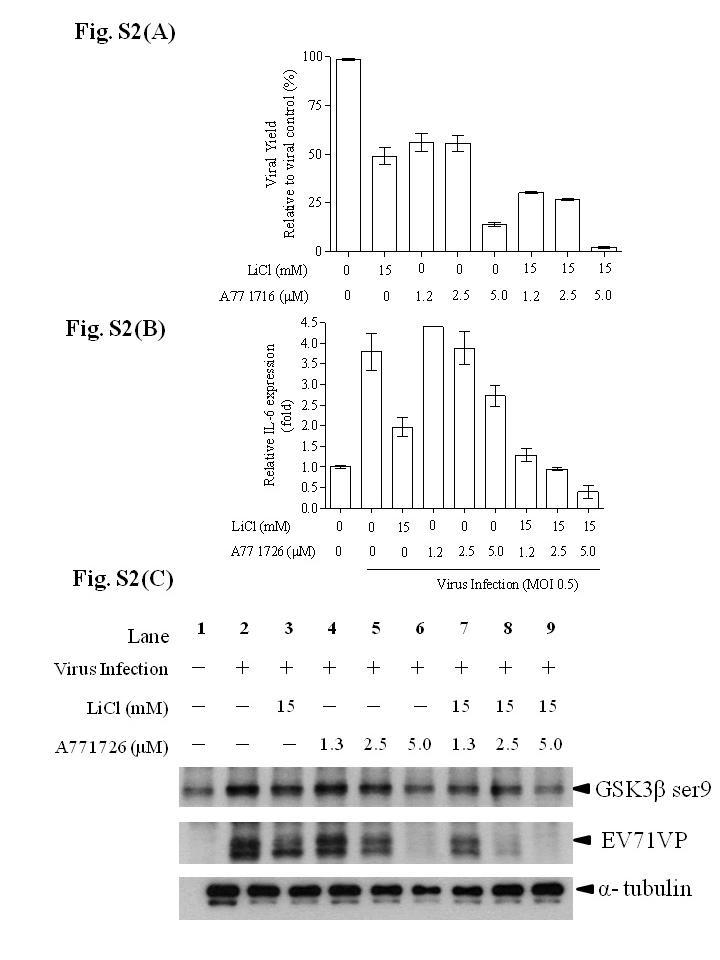

Supplement: Figure S2 — The effects of the combination of LiCl and A771726 on virus yield (A), IL-6 expression levels (B) and viral protein synthesis (C) in EV-A71-infected SF268 cells. SF268 cells were infected with EV-A71 (moi 0.5), and various concentrations of LiCl and A771726 were added to the infected cells for 48 h after infection. Assays were performed as described in the materials and methods section. Data are the mean ± s.e.m. from at least three parallel measurements per experiment. Symbol indicates significant differences as determined by one way ANOVA: *P<0.05, **P<0.001 *: compared to the percent of infected cells by treatment with A77172. (TIF) [file pone.0111331.s002.tif]

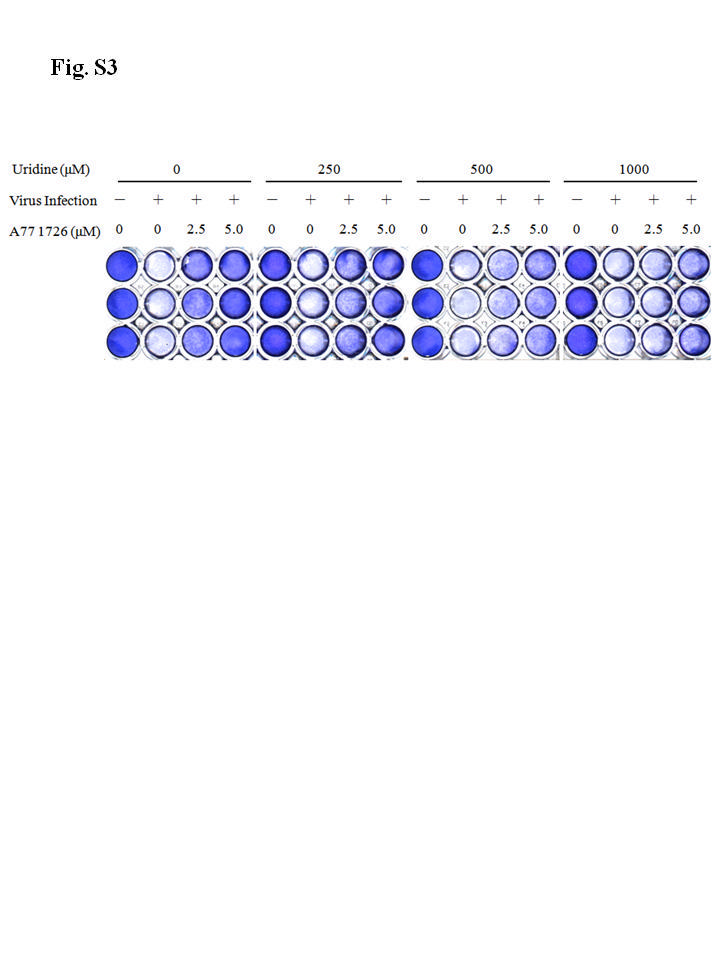

Supplement: Figure S3 — Inhibitory effects of A771726 on EV71-induced CPEs and the reversal of EV71-induced CPEs by uridine. Cells were infected with EV-A71 (moi 0.5), and various concentrations of A771726 and uridine were added to the infected cells. SF268 cells were all lysed at 72 h after EV-A71 infection, as shown in the VC (virus control) column. Measurements were made in three independent experiments. (TIF) [file pone.0111331.s003.tif]
